# Mechanochemically Scaled-Up Alpha Cyclodextrin Nanosponges: Their Safety and Effectiveness as Ethylene Scavenger

**DOI:** 10.3390/nano12172900

**Published:** 2022-08-23

**Authors:** David Rupérez, Nicolás Gracia-Vallés, Eva Clavero, Filomena Silva, Cristina Nerín

**Affiliations:** 1 I3A—Aragon Institute of Engineering Research, University of Zaragoza, 50018 Zaragoza, Spain; 2 ARAID—Agencia Aragonesa para la Investigación y el Desarrollo, 50018 Zaragoza, Spain; 3 Faculty of Veterinary Medicine, University of Zaragoza, 50013 Zaragoza, Spain

**Keywords:** food-grade α-cyclodextrin nanosponges, ethylene scavenger, HPLC-DAD, ball milling, imidazole, scale-up, active packaging

## Abstract

Aiming at the development of a greener ethylene removal alternative, the goal of this study was to scale up and ensure the safety of α-cyclodextrin nanosponges (α-CD-NS) for further use as ethylene scavengers. The solvent-free synthesis of α-CD-NS was successfully scaled up using α-cyclodextrin and N,N′-carbonyldiimidazole as cross-linkers (1:4 molar ratio) by means of mechanical alloying using a PM 100 ball mill by focusing on varying the rotation frequency, as determined by FTIR-ATR, X-ray diffraction, and TGA. α-CD-NS washing optimization was performed in water by monitoring the imidazole concentration in the washing solution through the validation of a fast and sensitive HPLC-DAD method. After 6 h at 40 °C, all imidazole was extracted, allowing a faster and less energy-dependent extraction. α-CD-NS absorbent capacity and porosity were also evaluated through BET isotherms and ethylene absorption experiments using α-CD-NS and commercially available absorbents (zeolite and bentonite) were performed by means of gas chromatography (GC) coupled to a flame ionization detector (FID). With a 93 µL h^−1^ kg_adsorbent_^−1^ ethylene removal capacity, α-CD-NS revealed the best ethylene scavenging activity when compared to the other absorbents, opening the doors for a safer, innovative, and eco-friendlier ethylene removal active packaging.

## 1. Introduction

In an increasingly globalized world, good resource management is of paramount importance. When it comes to food waste, figures can be alarming. The last Food Authority Organization (FAO) report [1] pointed out that an estimated 45 percent of fruits and vegetables are wasted annually [2], mainly due to early ripening. Ripening causes changes in fruit quality (color and firmness) and increases fruit and vegetable susceptibility to fungal contamination [3], which leads to food product (fruit/vegetable) loss [4]. The ripening process is mainly controlled by ethylene, also known as the plant growth hormone, which is a molecule responsible for numerous effects on the growth, development, and storage of fresh produces. It is produced by fruits, vegetables, and ornamental flowers, and it can have detrimental effects on their shelf-life even at μL L^−1^ concentrations [5]. The effects vary for each product, but some of the most common include decay, russet spotting, yellowing, odor, wilting and scalding, among others [6]. Therefore, new strategies to improve fruit and vegetable shelf-life are in demand, with extensive research taking place in the food packaging sector.

Since ethylene was shown to be a plant growth regulator just over 50 years ago [7], efforts have been made toward an effective ethylene removal approach. Several mechanisms have been studied, including adsorption on clays, zeolites, and different activated carbon nanoforms [8,9,10], oxidation using transition metals [11], photo-oxidation using UV lamps [12], and gaseous chemical inhibitors, such as 1-methylcyclopropene [13]. However, the most studied and used approach consists of chemical oxidation by means of potassium permanganate impregnated on high surface area materials such as clays [14]. This solution for ethylene removal is already being commercialized in the form of sachets, but due to the toxic nature of potassium permanganate, these sachets are very difficult to discard. Hence, new alternatives with safer and eco-friendlier ethylene removal compounds are needed.

Cyclodextrins [15] are enzymatically bio-synthetized from starch and can be defined as cyclic glucopyranose oligomers that have the ability to include a wide range of compounds, such as antimicrobials, essential oils, and gases within their cone-shaped lipophilic nano-cavity [16,17,18]. Cyclodextrin nanosponges (CD-NS) are a virtually non-toxic nanostructured type of cross-linked cyclodextrin with improved properties over cyclodextrin monomers because of their higher stability over a wide pH range (1–10) and temperatures of up to 130 °C [19].

Traditionally, CD-NS synthesis involves the use of high amounts of both energy and organic solvents [20]. Pedrazzo et al. [21] proposed a green synthesis using mechanochemistry that eliminates the use of organic solvents and uses only water for the washing step. Mechanochemistry is a solvent-free synthesis that relies on mechanical forces such as friction to transfer energy and form chemical bonds, reducing the energy requirements and aligning with the Green Chemistry Principles [22]. Additionally, a washing step is often necessary to remove any contaminants or subproducts that may be formed during the process. One of the benefits of mechanochemical synthesis is that it can be easily scalable. However, scaling up mechanochemical organic synthesis is still at an early age as the main industry focus of this technique has been on inorganic processes that are generally less temperature-dependent [23].

Trying to address the issue of food waste due to the early ripening of fruit and vegetables, the applicability of α-CD-NS for ethylene removal was studied. In this work, we aim to scale up the synthesis of a bio-based α-CD-NS, ensuring their food safety by validating an HPLC-DAD method to monitor imidazole, a toxic sub-product from the synthesis, and assessing the ethylene removal capacity of the final product by means of gas chromatography.

## 2. Materials and Methods

### 2.1. Materials

Alpha-cyclodextrin, N,N′-carbonyldiimidazole (CDI), imidazole, and ammonium dihydrogen phosphate were purchased from Sigma Aldrich (St. Louis, MO, USA). Milli Q water was generated using an Ultramatic Wasserlab purification system (Pamplona, Spain). For pH adjustment, 37% hydrochloric acid and 85% ortho-phosphoric acid (Scharlab, Madrid, Spain) were used. Analytical grade acetonitrile was purchased from Honeywell (Charlotte, NC, USA). Bentonites and zeolites were kindly provided by Nurel S.A. (Zaragoza, Spain).

### 2.2. Synthesis and Characterization of α-CD-NS

#### 2.2.1. Small Scale Synthesis

**Figure 1:**
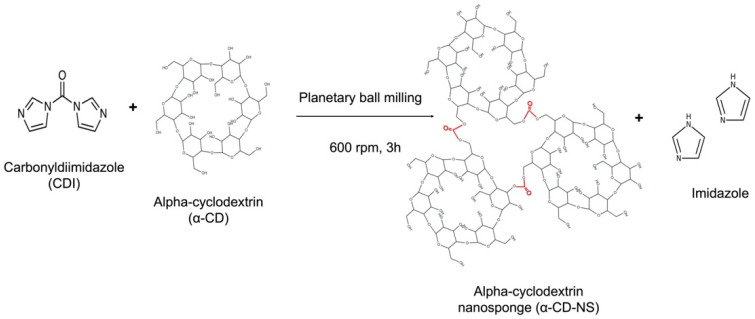
Cross-linking reaction of carbonyldiimidazole and α-cyclodextrin using ball milling, generating α-cyclodextrin nanosponges and free imidazole.

Following the synthesis described by Pedrazzo et al. [21], α-CD was dried in an oven at 100 °C until constant weight. The one-step, solvent-free synthesis was performed using a Retsch PM100 planetary ball mill (Haan, Germany) and CDI as a cross-linking agent in a 50 mL zirconium oxide jar with 10 zirconium oxide balls of 10 mm. The amount of α-CD and CDI to achieve the chosen 1:4 molar ratio was 3.38 and 2.25 g, respectively. After 3 h of 600 rpm sun wheel speed rotation, changing the direction of rotation every 15 min, the synthesis was completed (Figure 1). Once washed, the product was filtered on a Buchner funnel using a 0.2 µm mixed cellulose ester Whatman membrane filter (Maidstone, UK). Then, it was left to dry in an oven at 50 °C until a constant weight was achieved. Afterward, the dried α-CD-NS were stored in a desiccator to prevent moisture uptake.

#### 2.2.2. Synthesis Scale-Up

The synthesis was scaled up from a 50 mL to a 500 mL zirconium oxide jar. The diameter of the balls was kept at 10 mm, but the number of balls was increased to 100. The 1:4 α-CD and CDI ratio was identical, and the quantities used were 33.8 and 22.5 g of each, respectively. Different sun wheel speed rotation frequencies (310, 350, and 400 rpm) were tested, changing the rotation direction every 15 min. From here, the washing, filtering, and drying were performed identically as above.

#### 2.2.3. α-CD-NS Characterization

α-CD-NS characterization was performed using Fourier-Transform Infrared–Attenuated Total Reflectance (FTIR-ATR), Dynamic Light Scattering (DLS), thermogravimetric analysis (TGA), and X-ray diffraction (XRD). Prior to analysis, all of the samples were powdered and dried at 100 °C until a constant weight so that a consistent relative humidity between the samples can be assumed. FTIR-ATR spectroscopy was performed on a Jasco FT-IR 4100 (Madrid, Spain), and the samples were measured without further preparation. The spectrum was taken with 32 scans at 4 cm^−1^ resolution in a wavenumber range of 500–4000 cm^−1^. DLS analysis was carried out to obtain the hydrodynamic diameter and polydispersity index (PDI) of the washed α-CD-NS using a Brookhaven 90Plus DLS instrument (Holtsville, NY, USA). All of the measurements were conducted in Milli Q water at a concentration of 0.1 mg α-CD-NS mL^−1^ at 25 °C. A total of 10, 45 s runs were performed for each analysis. Thermogravimetric analyses were carried out on a Q5000SA analyzer from TA instruments (New Castle, DE, USA). Parameters were as follows: 50 mL min^−1^ synthetic air flow and ramp rate of 10 °C min^−1^ from room temperature to 800 °C. Powder X-ray diffraction was performed on a Panalytical Empyrean X-ray diffractometer from Malvern Panalytical (Malvern, UK). CuKα radiation (λ = 1.5419 Å) was used to scan the diffraction angles (2θ) between 5° and 60° at a speed of 4°/minute.

### 2.3. α-CD-NS Washing

#### 2.3.1. Influence of pH

The product of a small-scale α-CD-NS synthesis was divided in half and placed in two beakers containing 40 mL of deionized Milli Q water. Under constant stirring and temperature (25 °C), pH was monitored every hour using a Mettler Toledo pH meter (Greifensee, Switzerland). For one of the samples, concentrated hydrochloric acid was added, when necessary, to ensure the pH was kept below the pKa of imidazole (6.95). After eight hours, the solution was filtered, and the product was characterized as described in Section 2.2.3.

#### 2.3.2. α-CD-NS Washing Optimization

The same amount of product was washed under different conditions; 40 °C and 70 °C under constant stirring and ultrasonic extraction with different washing times (2, 4, 6, and 8 h) and two (14:1 and 28:1) water to α-CD-NS ratios.

To perform the experiments, 0.14 g of product were initially rinsed with 10 mL of Milli Q water to dissolve the unreacted CDI. The solution was then centrifuged at 4350 rpm using a VWR Mega Star 600R centrifuge (Radnor, PA, USA), and the supernatant was taken for further analysis. Then, the product was washed, changing only one of the above conditions at a time. At every time point, the solution was centrifuged using the same conditions as above, and the supernatant and pellet were kept for HPLC-DAD and nitrogen content analysis, respectively. The pellet was dried at 50 °C until a constant weight was obtained, and its nitrogen content was determined via elemental analysis on a Perkin Elmer 2400 Series II CHNS/O Organic Elemental Analyzer (Waltham, MA, USA) using optimum burning conditions and pure oxygen atmosphere.

### 2.4. HPLC-DAD for Imidazole Quantification

#### 2.4.1. Method Validation

An HPLC method for the determination of imidazole in the washing solution was developed based on the one described by You Zhu et al. [24]. Quantification was performed using an HPLC Waters 2695 Separations Module equipped with a Waters 2996 Photodiode Array Detector. Isocratic separation was performed using an Acquity UPLC BEH HILIC column (1.7 µm, 2.1 × 100 mm) from Waters (Milford, MA, USA). The composition of the mobile phase was a mixture of acetonitrile and 5 mM ammonium dihydrogen phosphate (80:20, *v*/*v*), adjusted with orto-phosphoric acid at pH 5. The mobile phase was filtered under vacuum using an Albet 0.2 µm nylon membrane filter (Dassel, Germany) and degassed for 30 min in an ultrasonic bath before use. An isocratic flow of 0.4 mL was applied, and the column oven was maintained at 35 °C. Standards were prepared gravimetrically using mobile phase as dilution solution. The run time was 5 min, and the wavelength defined to monitor imidazole at the retention time of 1.14 min was 215 nm. This method was validated according to the guidelines provided by the Food and Drug Administration (FDA) [25] and the International Conference on harmonization (ICH) [26], and the parameters studied were linearity, intermediate, intra- and inter-day precision and accuracy. Empower Pro software from Waters (Milford, MA, USA) was used for HPLC data analysis.

#### 2.4.2. Sample Quantification

After validation, imidazole was quantified in the supernatant samples (n = 3) from the different time points and conditions used in the washing optimization (see Section 2.3.2. for further details). When required, the samples were diluted using the mobile phase to fit within the method’s linear range and were analyzed as described above.

### 2.5. Evaluation of Ethylene Absorption Capacity of α-CD-NS and Other Absorbents

#### 2.5.1. Brunauer–Emmett–Teller (BET) Surface Area Analysis

N_2_ adsorption and desorption isotherms were recorded at −196 °C in an ASAP 2020 Micromeritics apparatus (Norcross, GA, USA). Prior to analysis, the synthesized α-CD-NS were degassed at 110 °C under a vacuum.

#### 2.5.2. GC-FID Method for the Determination of Ethylene

A method for ethylene analysis was developed by slightly modifying the one described by A. C. Guerreiro et al. [27]. Ethylene was measured using an Agilent 8860 gas chromatography system (Santa Clara, CA, USA) fitted with a TG-BOND Alumina (Na_2_SO_4_) column (0.53 mm internal diameter, 30 m length, and 10 µm film thickness) from Thermo Scientific (Waltham, MA, USA) and coupled to a flame ionization detector (FID) Separation was carried out in isocratic mode with a constant oven temperature of 60 °C. Inlet and detector temperatures were both 150 °C, and the run time was 5 min, with ethylene reaching the detector at 3.5 min. Helium gas was used as a carrier at a flow of 3.5 mL min^−1^. The gas samples were manually injected into the GC-FID using a Trajan 1 mL air-tight syringe fitted with a 50 mm length 0.63 mm OD side hole Luer-lock needle (Ringwood, VIC, Australia). A certified 10.29 µL L^−1^ gas mixture of ethylene balanced in synthetic air (Nippon gas, Belgium) was injected using different volumes (0.1–1 mL) on Splitless mode to achieve linear instrument response. OpenLab CDS ChemStation Edition C.01.10 software from Agilent Technologies (Santa Clara, CA, USA) was used for data analysis.

#### 2.5.3. Ethylene Removal Experiments

All of the ethylene removal experiments were carried out at room temperature (25 °C) and for a period of eight days. A series of absorbent compounds were tested for ethylene removal capacity: previously synthetized α-CD-NS and commercial bentonites and zeolites. After drying at 100 °C for 18 h, 0.3 g of each compound were placed inside 20 mL vials and closed with N 24 PP screw caps fitted with a 3.2 mm Mackerey-Nagel Silicone/PTFE septum (Allentown, PA, USA). Three replicates of a control vial without absorbent compound were also considered for every time point. Then, the inner atmosphere of the vials was replaced using the certified 10.29 µL L^−1^ ethylene gas mixture. For every time point replicate (24, 48, 72, 96, and 192 h), 0.5 mL were withdrawn through the septum using the air-tight syringe, injected manually, and analyzed using the method conditions described in Section 2.5.1. Experiments were conducted in triplicate.

### 2.6. Statistics

Data analysis was performed using Microsoft Excel software (Redmond, WA, USA), version 16.58. For variables with 3 or more categories, statistical analysis was performed using one-way ANOVA. A *p*-value indicating the probability of significance of <0.05 was used to indicate statistically significant differences.

## 3. Results

### 3.1. Synthesis Scale-Up and Characterization

Apart from the chemistry, there are a number of factors affecting the success of ball-milled organic synthesis [28], such as and by order of importance, rotation frequency, time, type and size of milling material, number of milling balls, and mode of operation.

α-CD-NS synthesis scale-up was performed using α-CD and N,N′-carbonyldiimidazole as a cross-linker with a 1:4 molar ratio in a 500 mL zirconium oxide jar with 100 10 mm diameter balls and 33.8 g of α-CD and 22.5 g of CDI. The reaction time was kept constant to focus on varying only the rotation frequency.

Three different rotation frequencies were tested: 310, 350, and 400 rpm. Above 400 rpm, jar temperature increased dramatically, which led to product degradation, and, therefore, rotations above 400 rpm were not tested.

**Figure 2:**
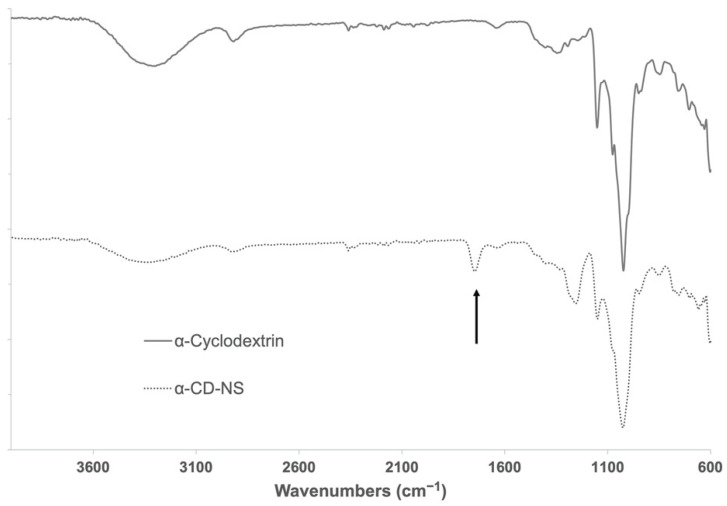
FTIR-ATR spectra of α-cyclodextrin and washed α-CD-NS. Arrow indicates the band of interest at around 1750 cm^−1^ assignable to the carbonyl group of the ester bond formed.

The cross-linked α-CD-NS polymers were insoluble in a range of solvents such as water, diethyl ether, dimethylformamide, petroleum ether, and dimethyl sulfoxide or ethanol, agreeing with previously reported data [20,21]. Figure 2 shows the FTIR-ATR spectra of α-CD compared to the synthesized α-CD-NS. The band present at around 1750 cm^−1^ indicated the presence of a carbonyl bond within the structure, which is indicative of an effective cross-linking between cyclodextrin hydroxyl groups.

**Figure 3:**
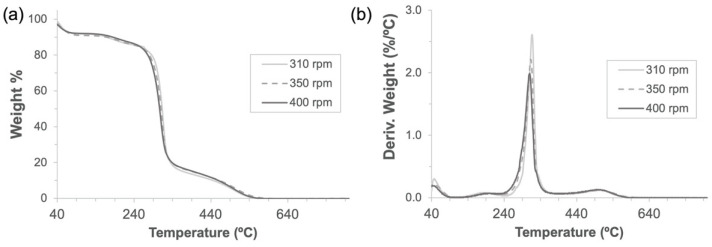
Thermogravimetric analyses (**a**) and their derivatives (**b**) of α-CD-NS obtained through 3 h of mechanochemical (ball mill) synthesis at 310, 350, and 400 rpm using a 500 mL zirconia jar.

As expected for mechanochemical synthesis [29,30], high yields (>95%) calculated by comparing the weight of the dry product against the sum of α-CD and one C=O bridge per cyclodextrin unit were obtained for all three frequencies tested. As can be seen by the thermogravimetric mass-loss curves and the corresponding derivative curves produced by all three rotation speeds (Figure 3), the synthesized polymers exhibited very close degradation paths with a relative maximum at 315 °C and, as a result, the same molecular structure was expected for them. It is worth pointing out that the three samples had very similar adsorbed water amounts, which were calculated to be 8.59 ± 0.56%.

**Figure 4:**
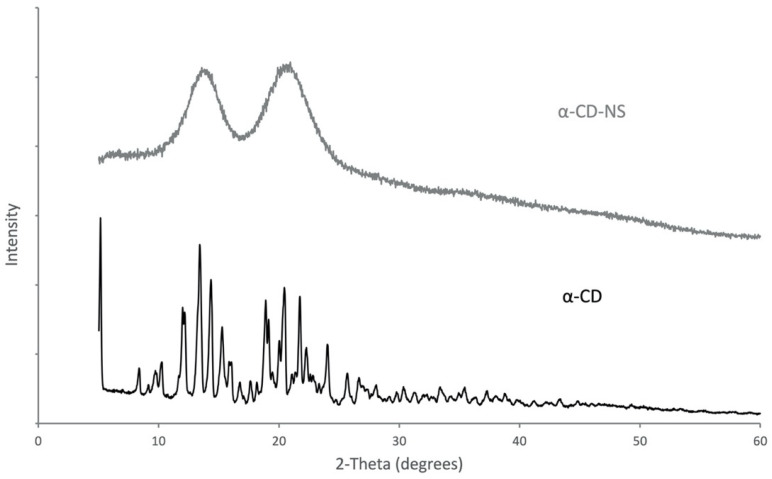
X-ray diffraction spectra for pure α-CD and the synthesized α-CD-NS.

A comparative analysis of the XRD diffraction patterns (Figure 4) was used to observe the differences in the spectra of α-cyclodextrin and the cross-linked α-cyclodextrin nanosponges. α-CD showed clearly defined reflections with sharp peaks, which confirmed its crystalline structure. However, the diffractogram of α-CD-NS had two wide peaks with maxima at 13.9° and 20.7°, which corresponded to an amorphous polymer structure. This phenomenon, which further characterizes α-CD-NS, has been observed for similar materials [31].

**Figure 5:**
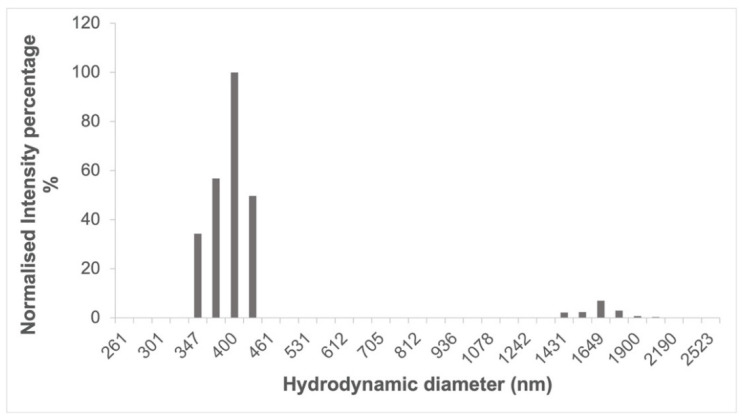
Size distribution of ball mill synthesized α-CD-NS after washing determined by dynamic light scattering (DLS).

After washing, the particle size of the cross-linked cyclodextrins (Figure 5) was around 400 nm as determined by DLS, having a polydispersity index of 0.222 ± 0.015, which is in line with what has been previously reported for cyclodextrin-based complexes after a wet milling cycle using a planetary ball mill [32]. In the case of cyclodextrin polymers that did not undergo a ball milling process, similar size measurements could only be achieved on the supernatant of a centrifuged dispersion [33].

**Figure 6:**
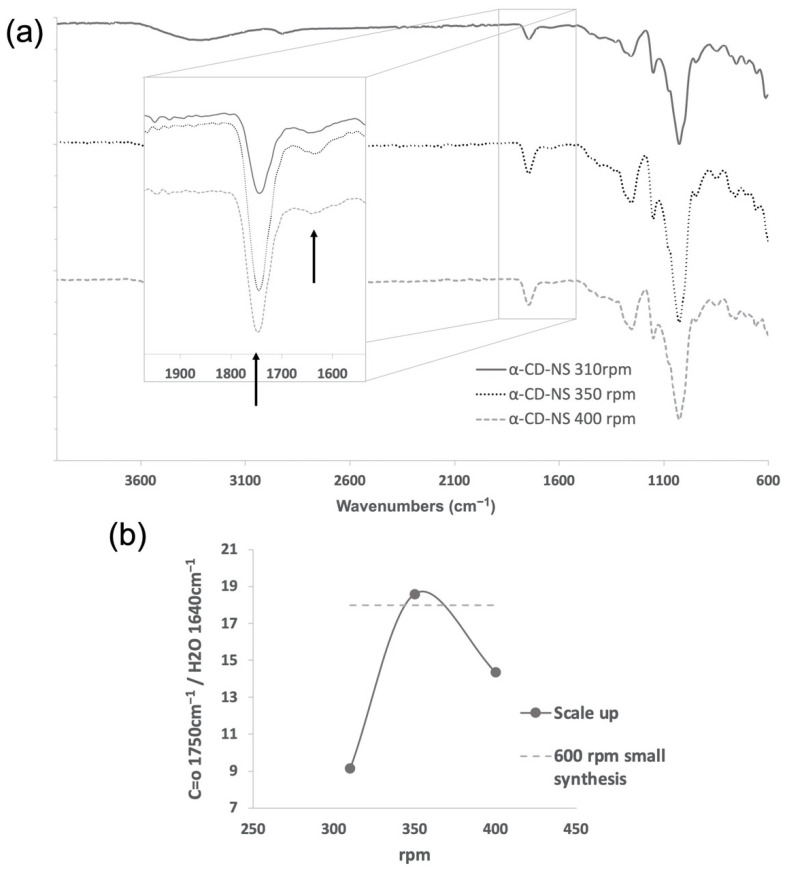
Analysis of α-CD-NS mechanochemical (ball mill) synthesis scale-up at 310, 350, and 400 rpm using a 500 mL zirconia jar: (**a**) FTIR-ATR spectra of α-CD-NS obtained with arrows indicating the band of interest at around 1750 cm^−1^ assignable to the carbonyl group of the ester bond and the band at 1640 cm^−1^ assignable to bending of the adsorbed water. (**b**) Carbonyl band (1750 cm^−1^) and water bending band (1640 cm^−1^) area ratio.

Figure 6a shows the FTIR-ATR spectra performed on the scaled-up α-CD-NS at the different rotations. As the TGA curves exhibited similar adsorbed water for all three samples, and to semi-quantitatively compare the intensity of the carbonyl band, hence determining differences in the cross-linking degree, the ratio of the area of the carbonyl band at 1740 cm^−1^ and the water bending band at 1640 cm^−1^ was calculated. Figure 6b shows the different ratios obtained for the rotation frequencies tested when compared to the ratio corresponding to the synthesis described by Pedrazo et al. [21] performed in a 50 mL jar at 600 rpm. We could observe that at 350 rpm, the carbonyl to water ratio was relatively higher than at 310 and 400 rpm. This could potentially mean a lower cross-linking degree at 310 rpm and some temperature-affected cross-linking degradation occurring at 400 rpm due to the sudden increase in reaction temperature.

Considering the high yield, TGA similarities, and the carbonyl-to-water ratio signal from FTIR-ATR, it could be stated that the 350 rpm rotation frequency produced an identical α-CD-NS product to the one obtained in the small-scale synthesis [21] and that the scale-up was successfully performed. Our results showed that the scale-up process cannot be performed by just proportionally increasing ball milling parameters and that close monitoring of the optimization is needed to ensure that the same final product is achieved while maintaining the yields.

### 3.2. HPLC-DAD Method Validation

**Figure 7:**
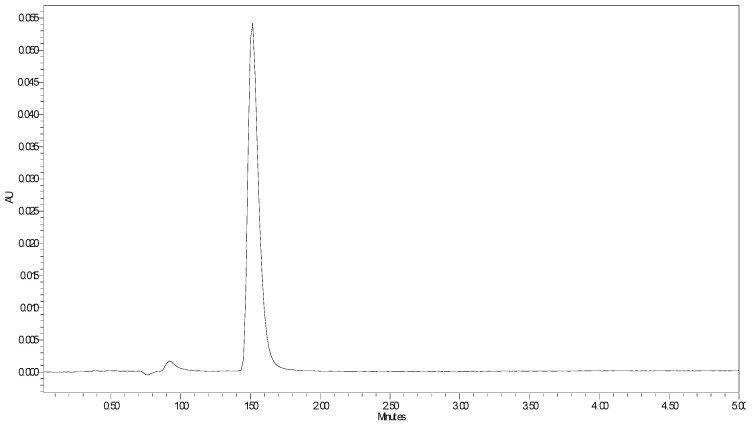
HPLC-DAD imidazole chromatogram at λ = 215 nm.

Several methods have been reported for the determination of imidazole derivatives using a different range of analytical techniques. However, none of them focus on just the determination of free imidazole. Zhu and collaborators reported a UV method for the determination of imidazole and two imidazole derivatives using high-performance liquid chromatography [24]. Considering this method as a starting point, we aimed at developing a faster, cheaper, more easily accessible, and precise method that would allow imidazole monitoring in α-CD-NS, even at very low concentrations using high-performance liquid chromatography (HPLC) coupled to a Diode Array Detector. In this way, the validated method will allow us not only to analyze imidazole in the synthetized α-CD-NS but also to evaluate a possible residual imidazole release when the α-CD-NS are applied as ethylene scavengers in contact with foods. The method was validated in terms of linearity, intermediate, intra- and interday precision, and accuracy, following a 5-day validation protocol according to FDA and ICH guidelines. The described chromatographic conditions produced a peak eluting at 1.48 min (Figure 7). To evaluate the method’s linearity, five replicates of eight evenly distributed calibration standards ranging from 0.1 to 10 µg g^−1^ were prepared gravimetrically and analyzed as described above. For intermediate precision evaluation, three quality control (QC) samples at low (LQC: 2 µg g^−1^), medium (MQC: 4 µg g^−1^), and high (HQC: 6 µg g^−1^) concentrations (n = 3) were also analyzed each day. Calibration curves were obtained by plotting peak area against concentration. The acceptance criteria involved a Pearson coefficient of at least 0.999 and the calibration standards’ accuracy within ±10%. To generate the best data for the chosen calibration range, six weighing factors (1/√x, 1/x, 1/x2, 1/√y, 1/y, 1/y2) were evaluated. Linearity data can be found in Table 1. The weighting factor 1/x was chosen since the sum of relative errors was smaller while still presenting a mean R^2^ value of at least 0.999. The limit of detection and quantification were calculated using a 25 ng g^−1^ standard and were 3.07 ng g^−1^ and 10.24 ng g^−1^, respectively, which is considerably lower than previously reported [24], meaning that very low concentrations of imidazole contaminant can be effectively detected in the finished product.

Interday precision and accuracy (Table 2) were evaluated at eight concentrations ranging from 0.1 to 10 µg g^−1^. The calculated relative standard deviations (RSD) were lower than 6% for all concentration levels, while accuracy was within a ± 10% interval from the target concentration. Intra-day precision and accuracy (Table 2) were assessed at four different concentration levels (0,1, 1, 5, and 10 µg g^−1^) using six replicates for each concentration. The obtained RSDs were lower than 5% for all concentrations, presenting an accuracy within a ± 10% interval. Moreover, intermediate precision and accuracy (Table 2) were assessed at three concentrations (2, 4, and 6 µg g^−1^) performed in triplicate over the 5-day validation protocol (n = 15). The results showed RSDs lower than 3% and the relative error in terms of accuracy being within a ± 2% interval.

Overall, since RSDs and accuracy for all criteria were lower than 15%, the HPLC-DAD method was successfully validated, allowing for robust and sensitive quantification of imidazole within 5 min.

### 3.3. α-CD-NS Washing Optimization

N,N’-carbonyldiimidazole (CDI) is a commonly used reagent in peptide synthesis for coupling amino acids and in organic chemistry for the formation of ester and amide bonds. In this work, CDI has been used to cross-link α-cyclodextrin units by means of ester formation via mechanochemical synthesis. After the reaction, a washing step is necessary to eliminate free imidazole generated in the reaction and to wash the unreacted CDI, and the imidazoyl carbonyl groups still present within the nanosponges network. Due to imidazole’s acute toxicity (LD_50_ in rat of 960 mg kg^−1^ bw) [34], the optimization of the washing step with water is needed to ensure its complete removal from the new material.

**Figure 8:**
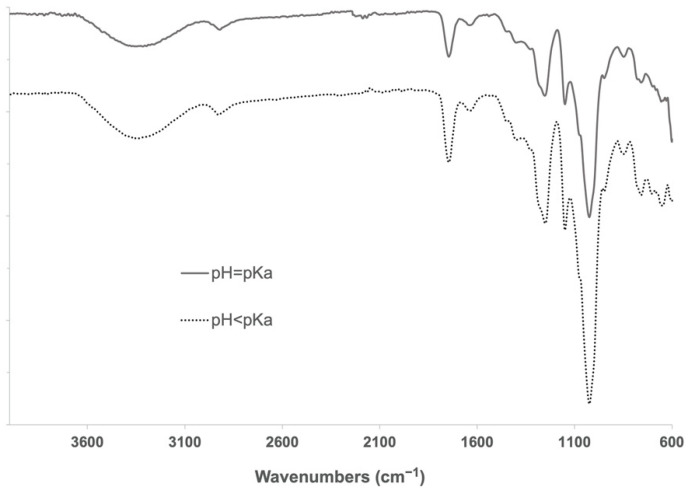
FTIR-ATR spectra of α-CD-NS washed at pH 6.9 (solid line) and pH 6.0 (dotted line). The band of interest at around 1750 cm^−1^ assignable to the carbonyl group was visible in both samples.

**Figure 9:**
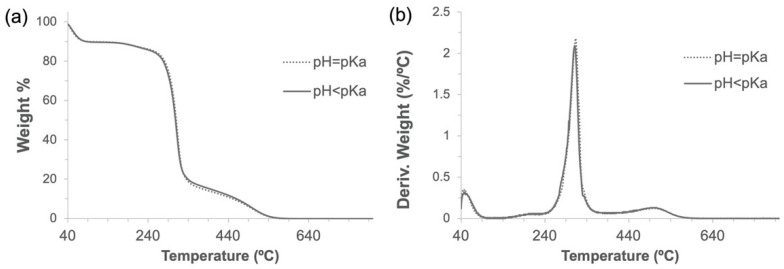
Thermogravimetric analyses (**a**) and their derivatives (**b**) of α-CD-NS washed at pH 6.9 and 6.0.

During the cross-linking reaction, CDI generates two molecules of imidazole, which are known to be able to catalyze the hydrolysis of ester bonds at different rates when being in its neutral form (imidazole pH = pKa) [35]. To determine a possible negative influence of pH in imidazole-induced hydrolysis of α-CD-NS, two washing protocols (8 h at 40 °C under constant stirring) at pH = pKa (neutral, reactive) and pH < pKa (protonated, unreactive) were compared. Two identical amounts of the synthesized product were washed, and their pH was monitored every hour. One of the washing solutions was maintained at a constant pH of 6.9, equal to imidazole’s pKa. To the other solution, concentrated hydrochloric acid was added when necessary to keep a constant pH of 6.0. After filtering and drying, the same yields (99.5%) were obtained. FTIR-ATR and TGA of the two washed products (Figure 8 and Figure 9) did not reveal any differences between the ratio of the area of the carbonyl band at 1750 cm^−1^ and the water bending band at 1640 cm^−1^, as well as in the thermogravimetric kinetics of the samples. This indicates that no molecular structure differences were produced during both washes at pH = pKa and pH < pKa, and, therefore, pH control is not required through the washing step.

**Figure 10:**
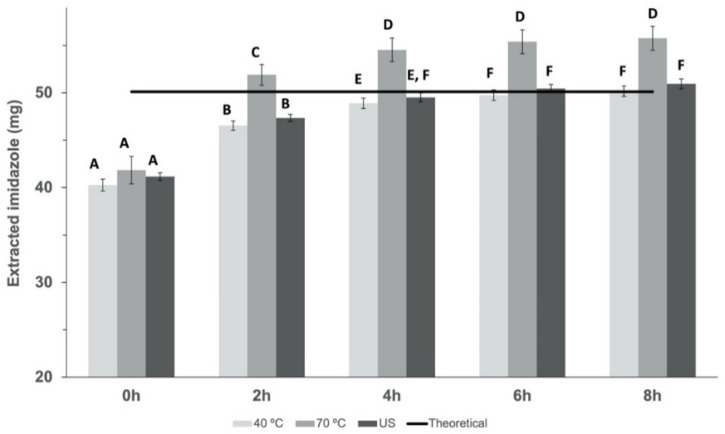
Results of the amount of imidazole extracted across time at 40 °C and 70 °C under constant stirring and ultrasonic extraction. Mean ± SD of three replicates is shown and compared against the theoretical imidazole in the samples. Different letters show significant differences (*p* ≤ 0.05) between samples.

**Figure 11:**
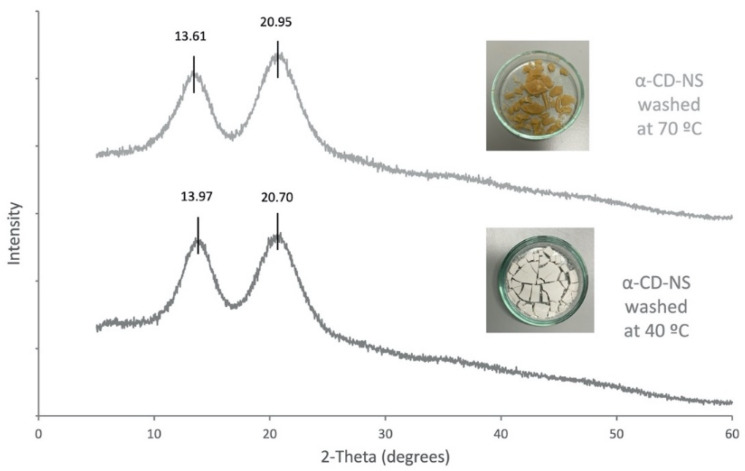
X-ray diffraction spectra α-CD-NS washed at 40 °C and at 70 °C.

The results obtained showed that there was no statistical difference (*p* > 0.05) among the different water-to-solute (α-CD-NS) ratios used. Figure 10 shows the imidazole extracted at different time points and conditions plotted against the theoretical imidazole present in the sample. Different letters show significant differences (*p* ≤ 0.05) between populations. We can observe that extraction by stirring at 70 °C gave the highest extraction efficiency, extracting up to 110% of the theoretical imidazole, which is within the method’s accuracy limits. However, after drying, a crystallized yellow product was obtained instead of the normal white powder (Figure 11).

As the α-CD-NS washed at 70 °C looked similar to a crystalized powder, an X-ray diffraction analysis was performed on α-CD-NS washed at 40 °C and 70 °C (Figure 11). Both diffractograms showed a disordered crystal structure corresponding to an amorphous structure, as already described in Figure 4. The X-ray diffractogram maxima obtained for the α-CD-NS washed at 40 °C (Figure 11) are the same as the ones obtained in Figure 4, indicating that the washing at 40 °C did not affect the αCD-NS amorphous structure. However, a subtle shift could be observed in the maximum of both broad peaks, suggesting a significant change within the amorphous structure, as the shifts were not all unidirectional.

As α-cyclodextrin is known to be present in different hydrate forms [36,37], we hypothesize that a type of cross-linked α-cyclodextrin hydrate is formed during washing at 70 °C, although more efforts are needed to fully characterize and understand these changes in the α-CD-NS structure.

According to our results, the extraction efficiency at 4 h cannot be considered significantly different (*p* < 0.05) between stirring at 40 °C or applying ultrasonic extraction. However, whereas for ultrasonic extraction, maximum efficiency (99.39 ± 0.96% imidazole extraction) was reached at 4 h, stirring at 40 °C only reached a maximum of extraction (99.39 ± 1.32%) at 6 h. Despite requiring longer processing times, a washing step for six hours at 40 °C under constant stirring were selected as the preferred washing conditions based on its easier scalability when compared to ultrasonic extraction. This was further confirmed by performing elemental analysis of the washed product targeting carbon, hydrogen, and nitrogen content, where a signal within the detection limit of the technique (±0.3% of Nitrogen) was considered negligible. Altogether, the described washing methodology allows for a faster washing step, with the potential to cut down energy costs.

### 3.4. α-CD-NS Ethylene Removal Capacity

**Figure 12:**
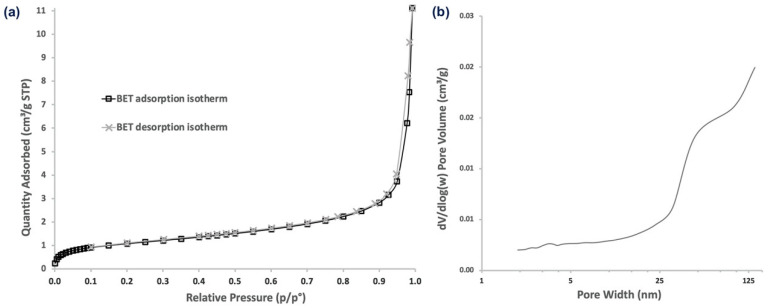
N_2_ adsorption/desorption isotherms (**a**) and pore size distribution (**b**) of α-CD-NS.

As α-CD-NS are formed by either the inner cavities of alpha-cyclodextrin or the outer cavities of the cross-linked network, we evaluated α-CD-NS porosity in an attempt to understand how ethylene could be absorbed by α-CD-NS. The porosities and pore sizes of α-CD-NS were examined by their N_2_ adsorption/desorption isotherms (Figure 12). The isotherms displayed a typical type II profile corresponding to the unrestricted monolayer-multilayer adsorption expected for non-porous or microporous adsorbent materials. The specific surface area was 3.90 m^2^ g^−1^, and the average pore diameter was 10.46 nm. The pore volume of pores less than 98 nm in width was 0.010 cm^3^ g^−1^. This high surface area match has been previously reported for other cyclodextrin monomers [38], suggesting polymerization does not have an effect on surface area increase. Moreover, the data suggest that the presence of macropores (>50 nm) within the cyclodextrin polymeric network would likely impede α-CD-NS from outperforming their monomer counterparts as ethylene scavengers if not for their higher physical and chemical stability and water insolubility, which is a major concern when developing products intended to be in contact with fruit, as they require high humidity environments (≥90% relative humidity).

Linear response using the GC-FID method described above was obtained, and the amount of ethylene inside the vials was successfully quantified by integrating the area under the ethylene peak. To evaluate the ethylene removal capacity, the ethylene ratio between each replicate and the control for each time point was calculated.

**Figure 13:**
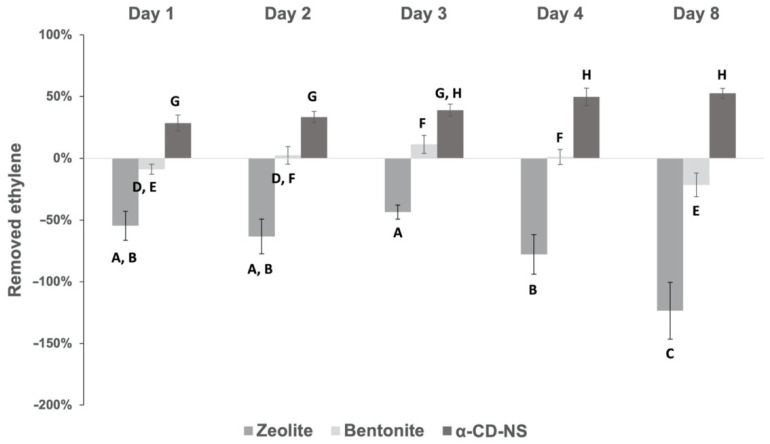
Ethylene removal percentage of α-CD-NS and commercial bentonite and zeolite across time. Mean ± SD of three replicates is shown. Different letters show significant differences (*p* ≤ 0.05) between samples.

Figure 13 shows the ethylene removal behavior of the different absorbent compounds tested. In the case of zeolites, the concentration inside the vial tended to increase throughout the assay, and, as a result, negative ethylene removal percentages ranging from −44 % to −124% were obtained. As zeolite’s adsorption capacity increases very rapidly with pressure [39], the results obtained can be due to the fact that zeolites already removed some ethylene during the vial filling process, which could later be released during the assay, meaning a fast, yet weak ethylene removal capacity. With ethylene removal percentages ranging from −22% to 11%, bentonites showed no clear behavior that could demonstrate any potential ethylene removal capacity. However, α-CD-NS showed a clear tendency as an effective ethylene removal across time. Analysis of variance (*p* > 0.05) showed that there was no difference between ethylene removed on day 3 and day 8, meaning that, after 72 h, the maximum α-CD-NS ethylene removal capacity was reached. Furthermore, ethylene concentration inside the vial was maintained until day 8, suggesting no release of the removed ethylene back into the vial’s atmosphere, which could indicate that an equilibrium state was reached [40], suggesting an ethylene removal kinetics similar to that of molecule encapsulation in cyclodextrins. α-CD-NS ethylene removal can be due to the effective encapsulation of ethylene gas molecules inside the α-CD cavity [41,42] and also due to its adsorption in the pores of α-CD-NS as other CD-NS have been described as effective gas carriers (oxygen) [43]. On day 3, α-CD-NS ethylene removal capacity was calculated as 18 mL of ethylene per kilogram of α-CD-NS (18 mL kg^−1^ of adsorbent), which corresponds to 93 µL of ethylene h^−1^ kg adsorbent^−1^. As most climacteric fruits and vegetables produce ethylene within the range of 0.1–10 µL kg^−1^ h^−1^ [44], α-CD-NS can be considered a suitable ethylene removal agent as it can remove more ethylene than what is produced by the fruit. When compared to permanganate-based ethylene scavengers, α-CD-NS ethylene removal performance, together with its non-toxic and organic, biodegradable nature, makes a reliable and effective ethylene removal alternative. Additionally, the fact that α-CD-NS does not react directly with ethylene makes it possible to develop a strategy to recover empty α-CD-NS and reuse this material, which cannot be undertaken in the case of permanganate-based ethylene scavengers, as permanganate readily reduces when in contact with ethylene.

## 4. Conclusions

Nowadays, the use of ethylene scavengers in the food packaging industry is often limited to different forms of potassium permanganate-based products, creating the need for a non-toxic, robust, environmentally friendly ethylene removal solution. In this work, a solvent-free, green synthesis of α-CD-NS was successfully scaled-up 10 times using α-cyclodextrin and N.N’-carbonyldiimidazole at a 1:4 (α-CD:CDI) molar ratio by mechanical alloying (3 h, 350 rpm rotation frequency) using a PM 100 planetary ball mill, as confirmed by FTIR-ATR, XRD, and TGA. A washing step with water was optimized by monitoring the presence of contaminants (unreactive CDI and imidazole) with validated liquid chromatography coupled to a diode array detector (HPLC-DAD) method targeting imidazole, allowing to achieve a high yield (>95%) contaminant-free α-CD-NS. Optimum washing conditions (99.39 ± 1.32% imidazole extraction) were reached at 6 h under constant stirring at 40 °C, with no pH monitoring required. To our knowledge, this is the first time that α-CD-NS are successfully scaled-up with a mechanochemistry method and the first time their ethylene scavenging properties are reported. With a 93 µL h^−1^ kg_adsorbent_^−1^ ethylene removal capacity, the synthesized α-CD-NS can be considered a green, biodegradable, and safe ethylene-scavenging alternative when compared to traditional potassium permanganate. Overall, this work demonstrates the potential of α-CD-NS to be a key player in the food packaging industry as an ethylene scavenger, helping towards better produce management and reducing fruit and vegetable losses throughout the food chain due to uncontrolled ripening.

## Figures and Tables

**Table 1:** HPLC-DAD imidazole linearity data. All concentrations are in µg/g. RSD, relative standard deviation. When applicable, values are presented as mean values ± standard deviation.

Weight	Linearity	Slope	Intercept	R^2^
1/x	0.1–10	54,545.60 ± 1303.41	1394.12 ± 987.53	0.9996 ± 0.0003

**Table 2:** Inter-day (n = 5), intra-day (n = 6) and intermediate (n = 15) precision and accuracy. All concentrations are in µg/g. RSD, relative standard deviation. When applicable, values are presented as mean values ± standard deviation.

	Theoretical Concentration (µg/g)	Measured (µg/g)	RSD (%)	Relative Error (%)
Inter-day n = 5	0.1	0.11 ± 0.01	5.64	5.95
	0.25	0.23 ± 0.01	4.11	−8.38
	0.5	0.54 ± 0.01	1.91	3.64
	1	0.98 ± 0.03	2.74	−3.46
	2.5	2.50 ± 0.03	1.02	−0.49
	5	5.08 ± 0.07	1.28	0.79
	7.5	7.55 ± 0.08	0.99	0.25
	10	10.03 ± 0.11	1.1	−0.24
Intra-day n = 6	0.1	0.12 ± 0.01	3.25	9.35
	1	1.00 ± 0.02	1.58	−0.93
	5	5.30 ± 0.14	2.55	4.92
	10	10.72 ± 0.29	2.68	6.16
Intermediate n = 15	2	2.08 ± 0.05	2.64	−0.10
	4	4.05 ± 0.10	2.43	0.61
	6	6.07 ± 0.09	1.55	1.12

## Data Availability

Not applicable.
